# Baseline microbiota of blueberries, soil, and irrigation water from blueberry farms located in three geographical regions

**DOI:** 10.1016/j.heliyon.2024.e40762

**Published:** 2024-11-29

**Authors:** Angelica Abdallah-Ruiz, Clara Esteban-Perez, Shecoya B. White, Wes Schilling, Xue Zhang, Eric T. Stafne, Alejandro Rodríguez-Magaña, Fernando Peña-Baracaldo, Carlos A. Moreno-Ortiz, Juan L. Silva

**Affiliations:** a Department of Biochemistry, Nutrition, and Health Promotion, Mississippi State University, Mississippi State, MS, 39762, USA; b Sequencing Department, Invitrocell SAS, Bogotá, 110111, Colombia; c South Branch Experiment Station, Coastal Research and Extension Center, Mississippi State University, Poplarville, MS, 39470, USA; d Facultad de Ciencias Económicas y Empresariales, Universidad Panamericana de Guadalajara, Guadalajara, 45010, Mexico; e Facultad de Ciencias Agropecuarias, Universidad de Ciencias Aplicadas y Ambientales U.D.C.A., Bogotá, 111166, Colombia; f Facultad de Ciencias Administrativas y Comerciales, Universidad de Ciencias Aplicadas y Ambientales U.D.C.A., Bogotá, 111166, Colombia

**Keywords:** 16S rRNA gene sequencing, Bacterial microbiota, Blueberries, Soil, Irrigation water

## Abstract

Bacterial microbiota was determined in fruit, soil, and irrigation water from blueberry (*Vaccinium* spp.) farms located in Cundinamarca, Colombia; Mississippi, United States; and Jalisco, Mexico. Bacterial communities were studied using 16S ribosomal ribonucleic acid (rRNA) gene amplification by targeting the V3–V4 hypervariable region. The most abundant phylum in fruit was Proteobacteria in Colombia and the United States and Firmicutes in Mexico. The most abundant phylum in soil and water was Proteobacteria for all regions. The top three genera found in fruit were *Heliorestis* (9.2 %), *Rhodanobacter* (3.3 %), and *Sphingomonas* (2.8 %) for Colombia, *Heliorestis* (23.1 %), *Thiomonas* (8.5 %), and *Methylobacterium* (3.3 %) for the United States, and *Heliorestis* (47.4 %), *Thiomonas* (9.1 %), and *Bacillus* (4.6 %) for Mexico. Colombia reported the highest (P*adj* < 0.05) alpha diversity for blueberries, and United States and Mexico had similar (P*adj* > 0.05) results. Beta diversity revealed bacterial communities in fruit differed (P < 0.05) by region. Bacterial differences existed between Colombia, United States, and Mexico for soil and fruit (P = 0.021, 0.003, and 0.006, respectively) and water and fruit (P = 0.003, 0.003, and 0.033, respectively). Blueberries grown in the three different regions have unique microbiota. Fruit and fruit-environment microbial composition also differed by region. These results provide a more complete profile of the bacterial communities on blueberries and their agricultural environments and could contribute to better management and decision-making practices in terms of plant health, food quality, and food safety.

## Highlights

• Proteobacteria were the most abundant phyla, except in fruit from Mexico.
• Two common fruit genera with abundance ≥2 percent were *Heliorestis* and *Thiomonas*.
• Alpha Diversity of fruit was similar between the United States and Mexico.
• Alpha Diversity varied between fruit, soil, and water in the United States and Mexico.
• Beta Diversity differed among fruit, soil, and water regardless of region.

## 1 Introduction

The microbial population in many fruits and vegetables is large and highly diverse [1], which affects food safety and quality, can create symbiotic relationships, and is associated with plant health [2,3]. Conventional microbiological analysis in blueberries commonly includes both aerobic and yeast and molds plate counts [4,5], which does not include specific microorganisms that might affect the safety and quality of blueberries. The accessibility to free-culture techniques, such as high-throughput sequencing, has contributed to the exploration of the rhizosphere and phyllosphere of many plants that are grown under various conditions [[6], [7], [8]]. In addition, these techniques can help detect microbial communities that might either support or inhibit the growth of pathogens [8,[9], [10]] as well as microorganisms that are used as biomarkers in plant protection strategies [3,11].

Colonized microbiota of crop plants is impacted by climatic factors (season, weather), agricultural inputs (fertilizers, pesticides), agricultural management practices (organic, conventional), agricultural environment (soil composition, irrigation water), and plant host species and genotype [[8], [12], [13], [14], [15]]. Gu et al. [16], reported that the relative abundance of some species in spinach (*Spinacia oleracea*) changed after irrigation with ground water showing an increase in *Dyadobacter* genus and *Flavobacterium succinicans*. Guron et al. [17], reported that lettuce (*Lactuca sativa*) and radishes (*Raphanus sativus*) grown in loamy sand soil had higher species richness than when grown in silty clay loam soil. Therefore, having a complete profile of the microbial ecology that is living on fresh produce and their growing environment, taking into consideration influential factors, can contribute to better management practices that impact plant health and fruit quality and safety.

The United States, Mexico, and Colombia all produce blueberries, with the United States being the largest producer [18], amounting to 648 million pounds in 2023 [19]. Mississippi, although not among the top-producing states, generates about 1.1 percent (3.3 million kg or 7.3 million pounds) of the total national production [20]. Mexico ranked number five in 2020 [18], becoming one of the main blueberry suppliers to the US market [21]. Colombia has increased production for both the domestic market and export. In 2020, Colombian export of blueberries increased 309 percent when compared to 2019 [22], with production mostly concentrated in the Cundinamarca and Boyacá regions.

Some studies using high-throughput sequencing have been applied to determine the microbiome of blueberries, their rhizosphere, and the soil where they are grown [7,23,24], a task that cannot be accomplished using conventional methods. The blueberry microbiome is influenced by factors such as the plant genotype, changes related to the domestication and breeding of the plant, soil properties, geographic location, among others [7,24]. The present study offers a new exploration into the microbial ecology associated with blueberries and their surrounding environment in three distinct regions, the United States, Mexico, and Colombia, addressing the potential influence of the country of origin on the microbiome structure. In addition, the present study might provide a better understanding of which components of agroecology (irrigation water and soil) represent the highest risk for pathogen contamination and/or opportunities to improve plant health. In addition, dominant microbiota in produce can help elucidate if agricultural practices or specific regions can either support or suppress the growth of plant and human pathogens. Therefore, the objective of this study was to investigate and compare the bacterial microbiota associated with fruit (blueberries), soil, and irrigation water of blueberry farms from three different regions: Cundinamarca, Colombia; Mississippi, United States; and Jalisco, Mexico.

## 2 Materials and methods

### 2.1 Sample collection

Samples were collected between May and September 2020 from blueberry farms located in Cundinamarca, Colombia (Latitude 4.8719992, Longitude −74.1449702); Jalisco, Mexico (Latitude 20.6720375, Longitude −103.338396); and Southern Mississippi, United States (Latitude 31.153317, Longitude −88.9830577). Blueberry samples were collected with sterile gloves at five different locations in a field of approximately 2500 m^2^ (the four corners and the middle) and placed into sterile bags (approximately 150 g from each location to make a composite sample). Selection of blueberries was based on the following criteria: maturity estimated by visual assessment (ripe berries: dark blue in color with smooth skin on the fruit) and no visible wounds, cuts or bruises. Blueberry varieties collected included Biloxi and Sharpblue in Cundinamarca, different Rabbiteye varieties in Mississippi, and Biloxi in Jalisco.

Soil samples were taken with sterile gloves and placed into sterile bags. The samples (approximately 150 g from each location to make a composite sample) were collected around the harvested blueberry plants. The soil surface layer was removed to take samples from tightly attached soil. Water samples (1 L each) were collected directly from the irrigation water source using sterile bottles. For well water, the faucet was cleaned with ethanol (70 %) and the water was allowed to run for 2 min before taking the sample. All samples were stored on ice and transported to the laboratory at the specified location. Samples were then evaluated by amplifying the 16S rRNA gene V3-V4 hypervariable region [8] using an iSeq 100 Illumina sequencer (San Diego, CA, USA) to determine the bacterial microflora background of the samples (fruit, soil, irrigation water) from the specified regions. Eight samples of fruit, soil, and water were taken from each region.

### 2.2 Sample preparation and DNA extraction

Blueberry samples were blended with a sterile mortar and pestle and homogenized in a stomacher (Stomacher 400 Circulator, Seward, Cincinnati, OH, USA) for 1 min. Four grams of each homogenized sample was centrifuged at 15000 *g* for 10 min at 4 °C (Model Sorvall Lynx 4000 Centrifuge, Thermo Scientific, Asheville, NC, USA) and the pellet was resuspended in a 4 ml DNA/RNA shield (Zymo Research, Irvine, CA, USA) to stabilize the nucleic acids.

Soil samples were agitated for 1 min by hand and then 2 g of sample was transferred to a sterile tube containing 4 ml of DNA/RNA shield (Zymo Research, Irvine, CA, USA). Water samples were agitated for 1 min by hand. For each sample, 500 μl was filtered through a 0.45 μm Fisherbrand™ Water-Testing membrane filter (Fisher Scientific, Pittsburgh, PA, USA) and then through a 0.22 μm Fisherbrand™ Water-Testing membrane filter (Fisher Scientific, Pittsburgh, PA, USA). The filter was aseptically divided into pieces and stored in sterile tubes containing 2 ml of DNA/RNA shield (Zymo Research, Irvine, CA, USA). All prepared samples were vortexed for 1 min and then stored at 4 °C until DNA was extracted.

Samples were vortexed and centrifuged prior to DNA extraction. The supernatant was partially discarded leaving 2 ml with the pellet that was resuspended in DNA/RNA shield. The DNA extraction was carried out using 1 ml of the prepared sample using Zymo DNA Miniprep Kits (Zymo Research, Irvine, CA, USA) according to the manufacturer's protocol. The samples stored in the DNA/RNA shield were transferred to ZR BashingBead™ Lysis tubes, shaken in a bead beater at 1500 rpm for 15 min, and centrifuged at 10000 g for 1 min. The supernatant (400 μl) was transferred to a Zymo-Spin™ III-F Filter in a collection tube for a second centrifugation at 8000*g* for 1 min. The filtrate was mixed with 1200 μl of ZymoBIOMICS™ DNA Binding buffer. Afterwards, the mixture was transferred to a Zymo-Spin™ IICR Column and centrifuged at 10000 g for 1 min. The column was washed 3 times with buffer solution, and the DNA was eluted with 50 μl ZymoBIOMICS™ DNase/RNase free water. The eluted DNA was transferred to a prepared Zymo-Spin™ III-HRC filter and centrifuged at 16000 g for 3 min. The resulting eluted DNA was quantified using a Quantus Fluorometer (Promega, Madison, WI, USA) and standardized to 5 ng/μl [25].

### 2.3 16S rRNA gene amplification, library preparation and next generation sequencing

The standardized DNA template was used to amplify the V3–V4 hypervariable region of the 16S rRNA gene. The targeted amplicon size of this hypervariable region is 460 bp approximately [26]. This region has been previously used in other 16S rRNA gene sequencing studies [27,28]. The region was amplified using the following primer set: Forward Primer = 5′ TCGTCGGCAGCGTCAGATGTGTATAAGAGACAGCCTACGGGNGGCWGCAG; Reverse Primer = 5′ GTCTCGTGGGCTCGGAGATGTGTATAAGAGACAGGACTACHVGGGTATCTAATCC. The PCR mixture contained 2.5 μl of DNA template (5 ng/μl), 5 μl of 1 μM PCR forward primer, 5 μl of 1 μM PCR reverse primer, and 12.5 μl of 2x KAPA HiFi Hot Start Ready Mix The PCR reaction was conducted using the following conditions: 3 min at 95 °C followed by 25 cycles of 30 s at 95 °C, 30 s at 55 °C, 30 s at 72 °C, and a final extension of 5 min at 72 °C (supplier's conditions, Illumina, San Diego, CA, USA). The amplifications were carried out in a thermocycler (Eppendorf, New York, NY, USA), and the PCR products were confirmed by electrophoresis (agarose gel 1.4 %) and photographed under UV light.

The resulting PCR products were purified using AMPure XP beads to separate the 16S rRNA V3 and V4 amplicon. Secondary PCR was carried out to attach dual indices and the sequencing adapters to the amplicons using the Nextera XT Index Kit (Illumina, San Diego, CA, USA). The reaction mixture contained 5 μl of purified PCR products, 5 μl of Nextera XT Index Primer 1 (N7xx), 5 μl Nextera XT Index Primer 2 (S5xx), 25 μl 2x KAPA HiFi Hot Start Ready Mix, and 10 μl of PCR grade water, under similar conditions as previously mentioned but using 8 cycles per run. The second PCR product was cleaned by AMPure XP beads and quantified by using a Quantus Fluorometer (Promega, Madison, WI, USA). Each library was diluted to 4 nM and 5 μl from each were added into one vial. Twenty microliters of the pooled sample (1 nM) contained up to 20 libraries was transferred to the cartridge that was placed in the Illumina ISeq100 platform (San Diego, CA, USA) for sequencing (2 × 150 bp). Two sequencing runs were used to include the 24 libraries for each region.

### 2.4 Data analysis

Raw sequencing data were demultiplexed using Illumina's bcl2fastq2 algorithm and qualified filtered using the Phred Quality Score (≥Q30). Primer sequences and reads with low quality scores were removed. The passed filter sequences were subjected to adapter trimming process. Clusters were assigned to samples, based on the cluster's index sequence. Clusters passing the Illumina chastity filter were used to generate the final filtered passed reads. Clusters passed the filter when no more than 1 base call in the first 25 cycles had a chastity of <0.6 [29]. Each cluster that passed the filter was converted to fastq files. Then, each fastq file was taxonomically classified using the Illumina-curated version of the Greengenes database [30,31] under the 16S Metagenomics App, version v1.1.1 from the Illumina BaseSpace Sequence platform. The algorithm that was used is a high-performance implementation of the Ribosomal Database Program (RDP) Classifier described by Wang et al. [32]. The sequence reads were classified by sample and taxonomic level from kingdom to genus.

The sequences were normalized by dividing the individual counts by the total number of counts per sample, so that the sum of the values was 1 [33]. The relative abundance based on phylum-level classification for fruit (blueberries), soil, and irrigation water samples was plotted and visualized as stacked bar charts utilizing R scripts. The data in the stacked bar charts includes ≥2 percent of the sequences in at least one sample within each group/country Alpha diversity was calculated, at the genus level, using the Shannon index with R package vegan and visualized as boxplots. Beta diversity was obtained, at the genus level, using the Bray-Curtis dissimilarity method and visualized by a Principal Coordinate Analysis (PCoA) plot using the R package ggplot2.

The Kruskal-Wallis test at a significance level of 0.05, followed by a pairwise comparisons using the Wilcoxon rank sum test that was adjusted with the Benjamini-Hochberg method using a corrected P-value <0.05 (P*adj* < 0.05), was used to compare the relative abundance and the alpha diversity of each group [34]. Permutational multivariate analysis of variance (PERMANOVA) was used to compare beta diversity among groups at a significance level of 0.05 [35].

## 3 Results and discussion

### 3.1 Bacterial communities associated with blueberries

**Fig. 1:**
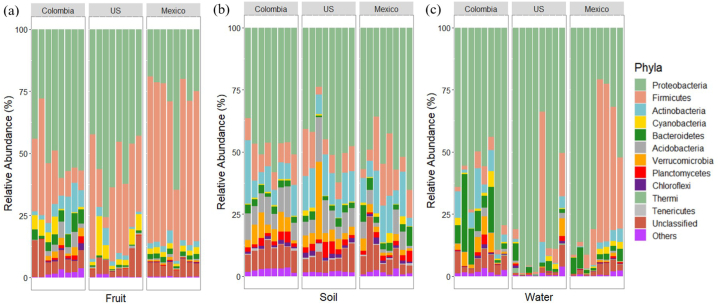
Relative abundance of bacterial taxa of fruit (a), soil (b), and irrigation water (c) by growing region. Data <2 percent in all samples is comprised in the “others" group.

The 16S rRNA gene V3-V4 region was sequenced to study the bacterial communities associated with blueberries. On average, 32601, 73332, and 84592 filtered passed reads were reported for each blueberry sample obtained in Colombia, Mexico, and the United States, respectively. Proteobacteria was more abundant (P*adj* < 0.05) in fruits from the United States and Colombia (54.4 % and 50.6 % respectively) than in fruits from Mexico (28.7 %), while Firmicutes was the most abundant (P*adj* < 0.05) phylum in fruits from Mexico (57.6 %) (Fig. 1a). Microbiome studies on grapes (*Vitis vinifera*) [36,37], muscadine (*Muscadinia rotundifolia* Michx.) [38], apples (*Malus pumila* Mill.) [15], tomatoes (*Solanum lycopersicum*), and peaches (*Prunus persica*) [36] have identified Proteobacteria and Firmicutes among the most predominant phyla in fruits, which suggests that they are endophytes to the plant microbiome. In this study, Proteobacteria also had a high relative abundance in the soil and water microbiome (Fig. 1b and c).

Relative abundance of Actinobacteria and Cyanobacteria were similar (P*adj* > 0.05) in all blueberries (2.1 %–6.4 %) among the three regions (Fig. 1a). Both phyla have been documented in fruits as endophytes [39,40] and Cyanobacteria have been associated with production of carotenoids [41]. Bacteroidetes in fruits from Colombia (>2 %) were more abundant (P*adj* < 0.05) than that found in the other regions.

At the genus level, both *Heliorestis* and *Thiomonas* were dominant among the blueberries grown in the three countries (Table 1). *Heliorestis* belong to a family group known as Heliobacteria. They have been isolated from paddy fields and other soils where their ability to form endospores may be related to their survival in these environments [42]. *Thiomonas,* as part of the Phylum Proteobacteria group, can use bioremediation to degrade different compounds, including arsenic to obtain energy [43]. *Thiomonas* can oxidize arsenite to arsenate which helps to detoxify the environment [44]. These two genera have been also detected in table olives as part of the initial microbiome before fermentation occurs [45].

**Table 1:** Main taxonomic genera (relative abundance ≥2 %) found in the bacteria community of fruit (blueberries), soil, and water samples from blueberry farms located in Colombia, the United States, and Mexico. Values represent means for the relative abundance (%).

**Fruit**		
**Colombia (%)**	**United States (%)**	**Mexico (%)**
*Heliorestis* (9.2)	*Heliorestis* (23.1)	*Heliorestis* (47.4)
*Rhodanobacter* (3.3)	*Thiomonas* (8.5)	*Thiomonas* (9.1)
*Sphingomonas* (2.8)	*Methylobacterium* (3.3)	*Bacillus* (4.6)
*Thiomonas* (2.0)		
*Rhodoplanes* (2.0)		

**Soil**		
**Colombia (%)**	**United States (%)**	**Mexico (%)**

*Candidatus Koribacter* (2.6)	*Rhodoplanes* (3.5)	*Sphingomonas* (3.5)
*Chthoniobacter* (2.1)	*Candidatus Koribacter* (2.2)	*Rhodoplanes* (2.3)
*Rhodoplanes* (2.1)	*Candidatus Solibacter* (2.1)	*Rhodanobacter* (2.2)
*Candidatus Solibacter* (2.1)	*Bradyrhizobium* (2.0)	
*Sphingomonas* (2.0)	*Edaphobacter* (2.0)	
*Azospirillum* (2.0)		
*Roseospira* (2.0)		

**Water**		
**Colombia (%)**	**United States (%)**	**Mexico (%)**

*Limnohabitans* (3.4)	*Pseudomonas* (12.0)	*Heliorestis* (22.0)
*Oxalobacter* (2.8)	*Curvibacter* (7.2)	*Acinetobacter* (7.3)
*Polynucleobacter* (2.2)	*Azohydromonas* (4.5)	*Thiomonas* (3.3)
*Paucibacter* (2.0)		*Bacillus* (2.1)

S*phingomonas* were predominant in blueberries from Colombia (Table 1). This genus represents a group of Gram-negative bacteria, belonging to the Proteobacteria phylum that is able to biodegrade hexachlorocyclohexane (HCH) and heavy metals [46,47]. In addition, *Sphingomonas* are associated with plant growth and enhanced resistance to abiotic stress [48]. *Sphingomonas* has also been isolated from other produce, such as tomatoes [8], apples (*Malus domestica*) [49], and grapes [50].

The *Methylobacterium* genus was predominant in fruits from the United States (Table 1). This genus has been detected on the surface of grapes [51] and can promote plant growth through production of cytokinins and auxin [52]. The *Bacillus* genus was predominant in Mexico (Table 1). *Bacilli* have been previously isolated from muscadines [38] and are considered biocontrol agents with antibiotic activity against certain human and plant pathogens [53,54]. Therefore, their presence could provide an unfavorable environment for human pathogen growth. Additionally, *Bacillus* have been associated with increased concentrations of phenolics, carotenoids, flavonoids and anthocyanins in strawberries (*Fragaria* × *annanasa*) [55].

### 3.2 Blueberry farm environment: bacterial communities associated with soil and irrigation water

The 16S rRNA gene V3-V4 region was sequenced to identify the bacterial communities associated with soil and irrigation water. On average, 23337, 30054, and 35742 filtered passed reads were obtained per soil sample collected in Colombia, Mexico, and the United States, respectively. Regarding irrigation water, 33064, 76586, and 57378 filtered passed reads were obtained from sample collected in Colombia, Mexico, and the United States, respectively.

The relative abundance based on phylum-level classification (Fig. 1b) indicated that Proteobacteria was the most abundant phylum in the soil in all of the countries (51.7 % for Mexico, 47.7 % for Colombia, and 45.4 % for the United States), followed by Firmicutes for Mexico and Colombia samples (12.2 % and 9.5 %, respectively) and Actinobacteria for the United States samples (12.4 %). These phyla have shown predominance in the soil microbiome in previous studies [56]. With respect to the water microbiome (Fig. 1c), Proteobacteria was the most abundant phylum in water for all countries (79.5 % for the United States, 64 % for Colombia, and 59.7 % for Mexico), followed by Firmicutes in Mexico and the United States (28.8 % and 10.5 %, respectively), and Actinobacteria in Colombia (6.4 %). These three phyla were predominant in the farming environments.

Firmicutes, Proteobacteria, and Actinobacteria are abundant in fertile soils that are rich in nutrients, such as carbon, nitrogen, phosphorus, and potassium [57]. These groups of bacteria contain 1-aminocyclopropane-1-carboxylate (ACC) deaminase enzymes, which have been associated with plant growth, less environmental stress, decreased flower wilting, and increased metal bioremediation [58,59]. Acidobacteria were also present at 9.1, 8.8, and 4.3 percent in relative abundance in soil samples of the United States, Colombia, and Mexico, respectively. These bacteria have been negatively associated with soils that are rich in carbon, leading to decreased presence of these bacteria in the soil [57]. Therefore, the organic resources present in soils could contribute to shape their microbial ecology. These phyla were previously identified as the most predominant in the rhizosphere soil of blueberry plants, regardless of the blueberry species [7].

Based on the most abundant genera found in soil samples, *Rhodoplanes* were detected among all countries and *Candidatus Koribacter* and *Candidatus Solibacter* were both detected in the United States and Colombia (Table 1). *Rhodoplanes* are involved in nitrogen cycling and contribute to both N-fixation and N-denitrification [60]. *Candidatus Solibacter* and *Candidatus Koribacter* have previously been isolated from soil [61] and are related to the carbon cycle [62] and sulfur metabolism [63]. Sulfur metabolism and the carbon cycle are important contributors to increasing soil acidity [64], plant growth, and postharvest fruit quality [65]. In addition to these contributions, soil microbiota is important in the humification and mineralization of organic matter in the soil. During humification, microorganisms transform organic matter into humic substances, which help to improve soil structure, water-holding capacity, and nutrient retention. During mineralization, they convert organic matter into inorganic nutrients that can be utilized by plants [66]. Hence, microbiota plays a role in maintaining the overall health of soil and plants. Soil genera with relative abundance ≥2 percent included *Bradyrhizobium* and *Edaphobacter* in the United States, *Chthoniobacter*, *Azospirillum*, and *Roseospira* in Colombia, and *Rhodanobacter* in Mexico (Table 1).

Each country had a distinctive microbiome in the irrigation water samples (Table 1). Predominant bacteria genera (*Heliorestis*, *Thiomonas*, and *Bacillus*) found in blueberries from Mexico were also found in water samples (Table 1). *Heliorestis* was found in water from this country; however, the bacteria have not been related to water microbiome [42]. Therefore, it might happen to be a microbial movement or transfer from another source to the water and from the water to the fruit.

Furthermore, the presence of certain microflora could influence the survival of human pathogens. *Pseudomonas* had a relative abundance of 12 percent in the water microbiome from the United States (Table 1). These bacteria have been previously detected in water [67,68] and are able to form biofilms that enhance *Listeria monocytogenes* colonization [9]. In addition, *Janthinobacterium*, which was found with high relative abundance (28 %) in 1 of 10 water samples from Colombia, has been positively correlated with the presence of *L. monocytogenes* in irrigation water that was used for tomatoes [69]. Therefore, presence of certain non-pathogenic microorganisms may indicate an increased likelihood of colonization for some pathogens.

In the present study, bacterial identification was detected at the genus level. While a species-level detection can offer deeper insights into individual microorganisms living within particular ecosystems, a genus-level detection is able to provide valuable information on bacteria groups that impact plant health, pathogen suppression, and fruit quality [8,70,71]. Bacteria sharing similar characteristics as part of being in the same genus have been identified as biocontrol and plant health strategies. For instance, the genera *Shingomonas* and *Methylobacterium*, which can be present in different parts of a plant, are associated with plant hormone promotion, and biotic stress protection [48,52]. In addition, several species of the genus *Bacillus* serve as plant growth promoters, antimicrobial producers, and pathogen suppressors [72]. In terms of fruit quality, bacteria spoilage is mainly associated with genera rather than specific species, such as *Lactobacillus*, *Erwinia*, *Leuconostoc*, *Pseudomonas*, *Gluconobacter*, *Acetobacter*, among others [73].

### 3.3 Alpha and beta diversity of blueberries, soil, and irrigation water by country

**Fig. 2:**
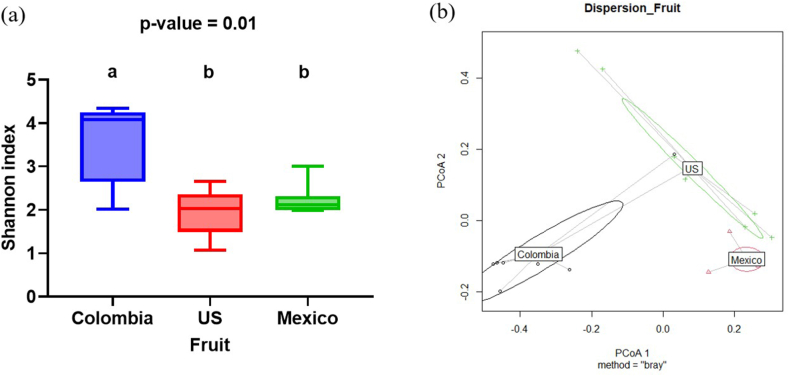
Alpha (a) and Beta (b) diversities in fruit among regions.

**Fig. 3:**
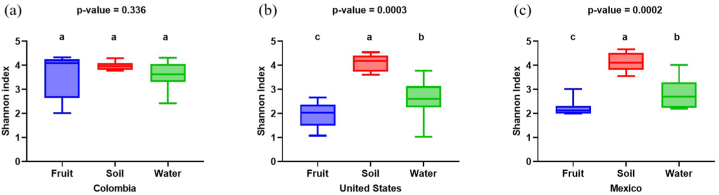
Alpha diversity of fruit, soil and irrigation water from blueberry farms located in Colombia (a), United States (b), and Mexico (c).

The Shannon index was calculated to determine the alpha diversity of fruit, soil, and water samples by geographical region (Colombia, United States, or Mexico group) at the genus level (Fig. 2, Fig. 3c). The index that was utilized accounted for richness and evenness of a single sample [74]. The Kruskal-Wallis test indicated that there were differences (P*adj* < 0.05) in alpha diversity among blueberry samples (Fig. 2a), suggesting that bacterial diversity in fruits within each growing region is different. Blueberries from the United States and Mexico showed similar (P*adj* > 0.05) patterns of microbial diversity, but these two showed lower (P*adj* < 0.05) alpha diversity than the Colombian blueberries. In addition, similar (P*adj* > 0.05) alpha diversity was obtained among fruit, soil and, water in Colombia (Fig. 3a). Mexico and the United States differed (P*adj* < 0.05) in alpha diversity among all samples, with the highest (P*adj* < 0.05) alpha diversity in the soil, followed by water, and then by fruit samples (Fig. 3b and c).

**Fig. 4:**
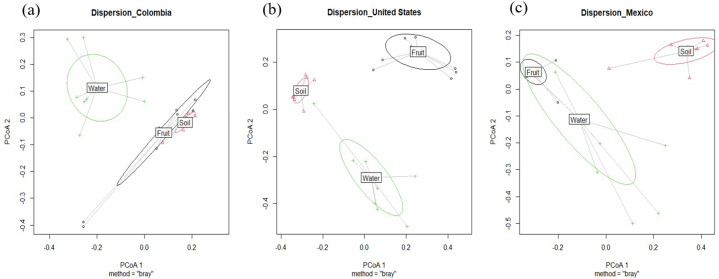
Beta diversity of fruit, soil and irrigation water from blueberry farms located in Colombia (a), United States (b), and Mexico (c).

The Bray-Curtis dissimilarity test was used to determine the beta diversity of fruit, soil, and irrigation water by growing region at the genus level (Fig. 2, Fig. 4c). PERMANOVA analysis indicated that the bacterial community diversity of blueberries varied among the growing regions (Fig. 2b). Differences in microbial community composition and distribution were detected in samples between Mexico and Colombia (P = 0.006), United States and Mexico (P = 0.003), and United States and Colombia (P = 0.015) (Fig. 2b). Within each region (Fig. 4a, b, and c), microbial profiles clustered by sample type, indicating bacterial diversity differences between soil and fruit (P < 0.05), water and soil (P < 0.05), and water and fruit (P < 0.05).

Blueberry varieties collected included Biloxi and Sharpblue in Cundinamarca, Colombia, different Rabbiteye varieties in Mississippi, United States, and Biloxi in Jalisco, Mexico (Table 2). Differences among varieties could contribute to the characteristic fruit microbiota by regions. Other studies have reported that the microbial diversity and richness of grapes and muscadine is affected by the cultivar [38,75]. In addition, environmental conditions inherent to each location (seasonal factors, air, human practices) impact the microbiota and this is indistinctive of plant species or fruit cultivar [38,76]. Temperature, rainfall, altitude, and sunlight may contribute to microbial community dissimilarities among regions (Table 2). Furthermore, production systems depict some differences. Blueberry farms visited in Jalisco have macro-tunnel production systems with a plastic covering used in the fields. In Mississippi, blueberries are grown in open fields where the fruit is directly exposed to the environment. In Colombia, some of the farms utilize anti-bird/anti-hail netting on the field and others have open fields. Therefore, a combination of factors likely has an impact on the microbial diversity found on blueberries, soil and irrigation water, where microorganisms might be adapting to specific conditions in the regions.

**Table 2:** Blueberry properties, soil characteristics, and weather patterns by geographical regions. Col: Cundinamarca, Colombia; US: Mississippi, United States; Mex: Jalisco, Mexico.

**Blueberry properties**		**Reference**	**Soil characteristics**		**Reference**	**Weather patterns**		**Reference**
**Acidity (citric acid %)**	Col: 0.72–0.95	[77]	**Classification**	Col: Andisols US: Alfisols and Ultisols Mex: Regosols	[78]	**Temperature (°C)**	Col: 8-14 US: varies according to seasons. 3 -15 (winter), 18–33 (summer) Mex: 15.24–24.5	[79]
	US: 1.0–1.6	[80]			[81]			[82]
	Mex: 1.17–1.22	[83]			[84]			[85]
**Soluble solids (°Brix)**	Col: 11-12	[77]	**Nutrients**	Col: Soil fertility is medium to high.	[78]	**Rainfall (mm)**	Col: 800-1400	[79]
	US: 12.3–13.6	[80]		US: Low organic matter	[81]		US: 1270-1651	[86]
	Mex: 13.2–13.9	[83]		Mex: Low organic matter	[84,87]		Mex: 720-2300	[85]
**pH**	Col: 3.19–3.32	[77]	**Water retention**	Col: High water retention.	[78,79]	**Altitude** **(msnm)**	Colombia: 2000-2600	[79]
	US: 2.9–3.5	[80]		US: High water retention.	[81]		US: ≤152.4	[88]
	Mex: 3.83	[83]		Mex: High water retention.	[84]		Mex: 800-2300	[85]
**Varieties**	Col: Biloxi and Sharpblue	[77], Col growers [89] US growers [83]	**pH**	Col: 4.8–5.5	[79]	**Average sunshine (hours/day)**	Col: 3.5–4.8	[79]
	US: Rabbiteye varieties			US: 4.6–5.5	[81]		US: 4.9–9.1	[82]
	Mex: Biloxi			Mex: 5.4	[90]		Mex: 4.5–6.4	[85]

## 4 Conclusions

Proteobacteria was identified as the most abundant phyla in all samples, except in blueberries from Mexico, where Firmicutes was the most abundant. Among genera with relative abundance ≥2 percent, *Heliorestis* and *Thiomonas* were commonly found in fruit and *Rhodoplanes* were commonly found in soil. No main genera were shared among regions for irrigation water. Alpha diversity associated with fruit was similar between the United States and Mexico. However, beta diversity revealed microbiota differences by fruit among all regions. In addition, beta diversity highly differed by sample type regardless of region. Our study expands the knowledge on the bacterial microbiota composition and diversity associated with blueberries and their surrounding environment (soil and water) of the studied regions. Future research could be directed to identify fungi diversity and their interaction with bacterial communities and to study the survival of specific taxa in fruit and soil associated with biocontrol (pathogen control) and plant growth.

## CRediT authorship contribution statement

**Angelica Abdallah-Ruiz:** Writing – review & editing, Writing – original draft, Validation, Project administration, Methodology, Investigation, Formal analysis, Conceptualization. **Clara Esteban-Perez:** Writing – review & editing, Validation, Methodology, Investigation, Formal analysis, Conceptualization. **Shecoya B. White:** Writing – review & editing, Resources, Methodology. **Wes Schilling:** Writing – review & editing, Methodology. **Xue Zhang:** Writing – review & editing. **Eric T. Stafne:** Writing – review & editing. **Alejandro Rodríguez-Magaña:** Writing – review & editing. **Fernando Peña-Baracaldo:** Writing – review & editing, Methodology. **Carlos A. Moreno-Ortiz:** Writing – review & editing, Validation, Resources, Conceptualization. **Juan L. Silva:** Writing – review & editing, Resources, Project administration, Methodology, Conceptualization.

## Data availability statement

Data will be made available on request.

## Declaration of competing interest

The authors declare that they have no known competing financial interests or personal relationships that could have appeared to influence the work reported in this paper.
